# Oscillation in Cycle Length Induces Transient Discordant and Steady-State Concordant Alternans in the Heart

**DOI:** 10.1371/journal.pone.0040477

**Published:** 2012-07-05

**Authors:** Seth H. Weinberg, Leslie Tung

**Affiliations:** Department of Biomedical Engineering, The Johns Hopkins University, Baltimore, Maryland, United States of America; Georgia State University, United States of America

## Abstract

Alternans is a beat-to-beat alternation of the cardiac action potential duration (APD) or intracellular calcium (Ca_i_) transient. In cardiac tissue, alternans can be spatially concordant or discordant, of which the latter has been shown to increase dispersion of repolarization and promote a substrate for initiation of ventricular fibrillation. Alternans has been studied almost exclusively under constant cycle length pacing conditions. However, heart rate varies greatly on a beat-by-beat basis in normal and pathological conditions. The purpose of this study was to determine if applying a repetitive but non-constant pacing pattern, specifically cycle length oscillation (CLO), promotes or suppresses a proarrhythmic substrate. We performed computational simulations and optical mapping experiments to investigate the potential consequences of CLO. In a single cell computational model, CLO induced APD and Ca_i_ alternans, which became “phase-matched” with the applied oscillation. As a consequence of the phase-matching, in one-dimensional cable simulations, neonatal rat ventricular myocyte monolayers, and isolated adult guinea pig hearts CLO could transiently induce spatial and electromechanical discordant alternans followed by a steady-state of concordance. Our results demonstrated that under certain conditions, CLO can initiate ventricular fibrillation in the isolated hearts. On the other hand, CLO can also exert an antiarrhythmic effect by converting an existing state of discordant alternans to concordant alternans.

## Introduction

Alternans is a beat-to-beat alternation of the action potential duration (APD) and/or intracellular calcium (Ca_i_) transient amplitude. At the cellular level, depending on the coupling between transmembrane voltage (V_m_) and Ca_i_, alternans can be electromechanically concordant in which long-short alternations in APD occur concurrently with large-small alternations in Ca_i_, or electromechanically discordant in which long-short APD alternans occur concurrently with small-large Ca_i_ alternations [1]. At a tissue level, alternans can be spatially concordant in which long-short alternations in APD or Ca_i_ are in-phase throughout the tissue, or spatially discordant in which long-short alternations are adjacent to short-long alternations separated by a nodal line [2], [3]. Spatially discordant alternans increases dispersion of refractoriness and creates a substrate for wavebreaks and initiation of ventricular fibrillation [3], and hence, is considered to be proarrhythmic.

Alternans has been studied extensively in the context of the restitution relationship for APD [1]. More recent studies have focused on the role of Ca_i_ dynamics and voltage-calcium coupling in promoting alternans [4], [5]. Alternans is typically studied under conditions of pacing at a constant cycle length (CL), i.e. at a fixed heart rate, even though heart rate varies on a beat-by-beat basis under normal physiological conditions, and indeed the absence of variability indicates a poor prognosis [6]. In this study, we used a cyclic pacing pattern, specifically oscillation in CL, to elicit alternans. Clinically, oscillation in heart rate has been observed in heart failure, post-myocardial infarction, or hypertensive patients [7]–[9], and has been found to immediately precede the onset of arrhythmia [9]. However, the linkage of this oscillation to arrhythmia initiation has not been previously investigated.

A few studies have considered non-constant CL pacing. Gilmour and colleagues showed that brief but specific sequences of precisely timed, irregularly spaced beats can promote fibrillation [10], [11]. Several studies have used feedback algorithms to vary CL in real-time to either control diastolic interval (DI) [12] or suppress alternans [13], [14]. Gauthier and colleagues showed that oscillation in CL induces and amplifies alternans amplitude at CLs near the alternans onset in an iterative map model [15], [16] and in frog hearts [17]. In our study, we demonstrate both the pro- and antiarrhythmic consequences induced by cycle length oscillation (CLO) in cardiac cells, tissue, and isolated hearts by utilizing simulations and optical mapping experiments that together encompass 0-, 1-, 2-, and 3-dimensional systems.

## Methods

### Ethics Statement

All research involving experimentation on vertebrate animals was approved by the Johns Hopkins Animal Care and Use Committee (IACUC Assurance #A3272-01). The methods of euthanasia are consistent with recommendations of the Panel on Euthanasia of the American Veterinary Medical Association. All efforts were made to minimize discomfort, distress, pain and injury.

An expanded methods section is provided in the online supplement (Text S1). In brief, single cell and one-dimensional cable simulations were performed using the Shiferaw-Sato-Karma ionic model [5], which integrates a detailed description of calcium cycling with a canine ionic model.

Constant pacing (pacing at a constant CL) was applied to the models for 50 or 51 beats, after which CLO, defined as pacing with CL:
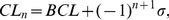
(1)was applied for an additional 50 beats. *BCL* is a constant basic cycle length, and *σ* is the amplitude of the applied oscillation, which was typically in the range of 5–40 ms (1–25% of *BCL*).

Our procedure to create and optically map cultured cell monolayers has been previously described [18]. Briefly, neonatal rat ventricular myocytes were enzymatically dissociated from 2-day old Sprague-Dawley rat hearts, plated to form confluent monolayers, and stained with the voltage-sensitive fluorescent dye, di-4-ANEPPS, or the calcium-sensitive fluorescent dye, Rhod-2-AM, and continuously superfused with Tyrode's solution. Contact fluorescent imaging was used to optically map the cell monolayers.

Our procedure for optically mapping isolated guinea pig hearts has been previously described [19]. Briefly, the hearts of Hartley guinea pigs (200–700 g) were excised, mounted on a Langendorff perfusion system, stained with di-4-ANEPPS, and immersed in a transparent plexiglass chamber filled with Tyrode's solution. A tandem-lens assembly, including two 150 W halogen lamps and a 100×100 pixel CMOS camera (Ultima-L, SciMedia, Costa Mesa, CA) was used to optically map the isolated hearts.

Cell monolayers or isolated hearts were paced using a platinum bipolar point electrode at a constant cycle length for 50 beats, after which CLO at a prescribed *σ* was applied as described above. Cell monolayers were paced near the cover slip edge, and isolated hearts were paced on the epicardial surface at the left ventricular base. Pacing sites are indicated in the spatial maps presented.

Individual signals recorded during optical mapping were processed, and the bipolar pseudo-electrocardiogram (pseudo-ECG) was computed as previously described [20]. Ca_i_ and APD alternans maps were computed by taking the difference of Ca_i_ transient amplitudes and APDs, respectively, on successive beats at each experimental recording site or discretized simulation location. Nodes and nodal lines were identified as regions of no alternans. Summary values were expressed as mean ± standard deviation. Statistical significance for paired comparisons of instances of ventricular fibrillation was determined using McNemar's Chi-squared test.

## Results

**Figure 1:**
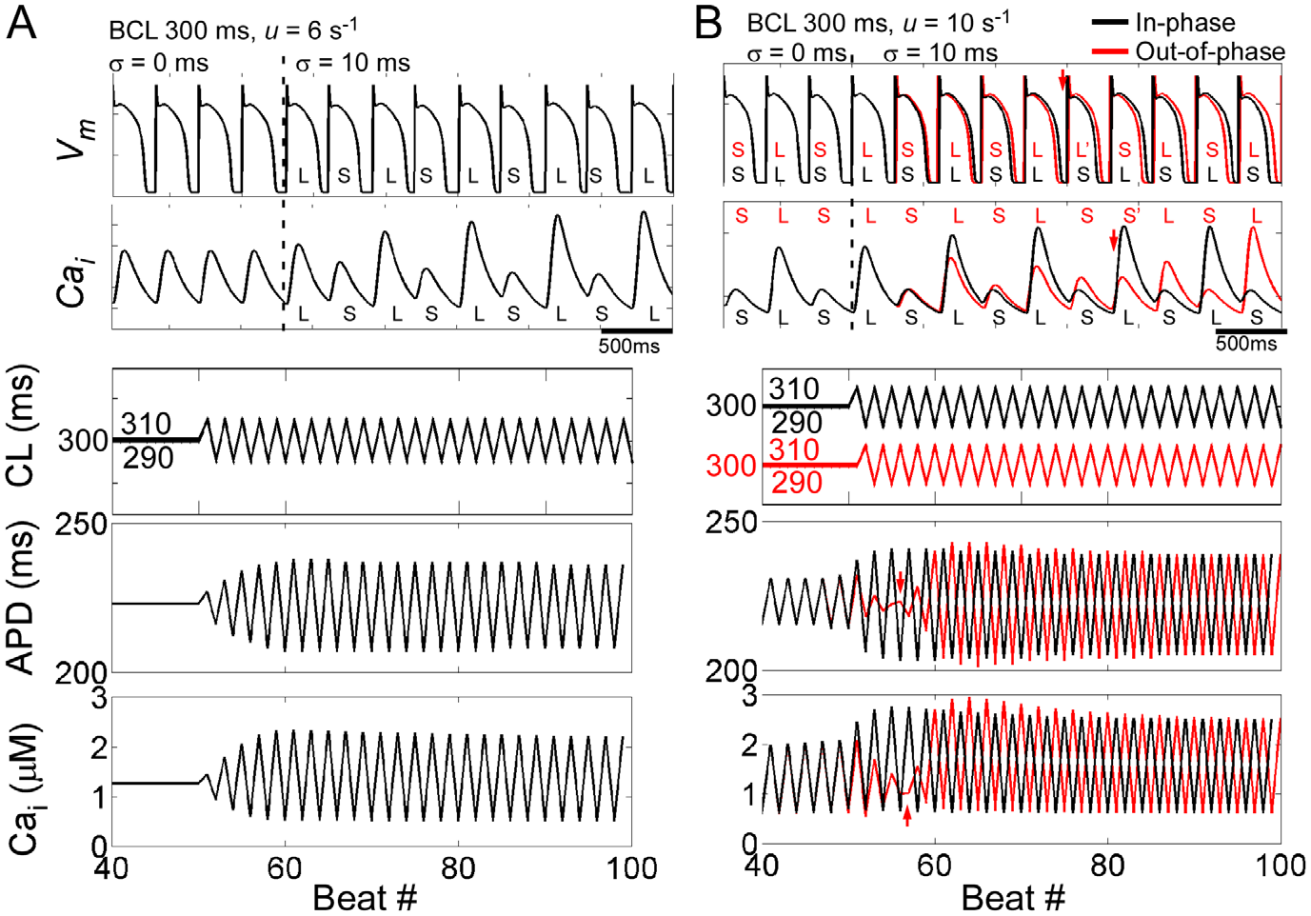
Cycle length oscillation in a single cell model. V_m_, Ca_i_, CL, APD, and Ca_i_ transient amplitude before and during CLO for *u* = 6 (**A**) and 10 (**B**) s^−1^. L and S indicate long and short APDs and large and small Ca_i_ transient amplitudes, respectively. In **B**, CLO applied in-phase (after 50 beats, black traces) and out-of-phase (after 51 beats, red traces) are shown. Phase reversal is indicated by a red arrow.

V_m_ and Ca_i_ traces before and during CLO from single myocyte simulations are shown in Figure 1. A parameter that modulates the sensitivity of sarcoplasmic reticulum (SR) calcium release to SR load, *u*, was set to 6 s^−1^. Qualitatively, we can understand the induction of alternans during CLO simply due to APD restitution, where APD is a monotonically increasing function of the preceding DI, by the following: 1. The initial step to a longer CL (*BCL*+*σ*) prolonged the DI and subsequent APD. 2. The following shorter CL (*BCL*−*σ*), combined with the prolonged APD, synergistically acted to shorten the next DI and subsequent APD. 3. The shorter APD, combined with the next longer CL, in turn prolonged the next DI and subsequent APD even further. The amplification continues until a new steady-state alternation was reached (Fig. 1A), consistent with previous studies [15]–[17]. Our simulations demonstrated that Ca_i_ alternans similarly grew in amplitude until reaching a steady-state.

### Influence of onset phase of CLO: cell simulations

We then investigated the consequence of applying CLO when alternans was already present following constant pacing. By increasing *u* from 6 to 10 s^−1^, alternans was induced for constant pacing at *BCL* of 300 ms (first 3 beats of Fig. 1B). When CLO was applied “in-phase”, meaning the first “long” CL (*BCL*+*σ*) was applied following a “short” APD, the “long” DI was prolonged further, and alternans amplitude grew and reached a new steady-state alternation after several beats (Fig. 1B black traces).

When the initial APD alternation and CLO were “out-of-phase”, the first “long” CL prolonged the “short” DI, resulting in a slightly longer “short” APD (Fig. 1B red traces). This pattern was reversed on the next beat, dampening the alternans amplitude until a phase-reversal occurred (red arrows), after which the alternation and CLO became “phase-matched” and increased in amplitude until reaching a steady-state. Additionally, the APD and Ca_i_ phase reversal occurred a beat apart, inducing transient electromechanical discordant alternans. Importantly, APD and Ca_i_ alternans amplitude reached the same steady-state value, regardless of the initial phase.

### Influence of spatial scale: cable simulations

**Figure 2:**
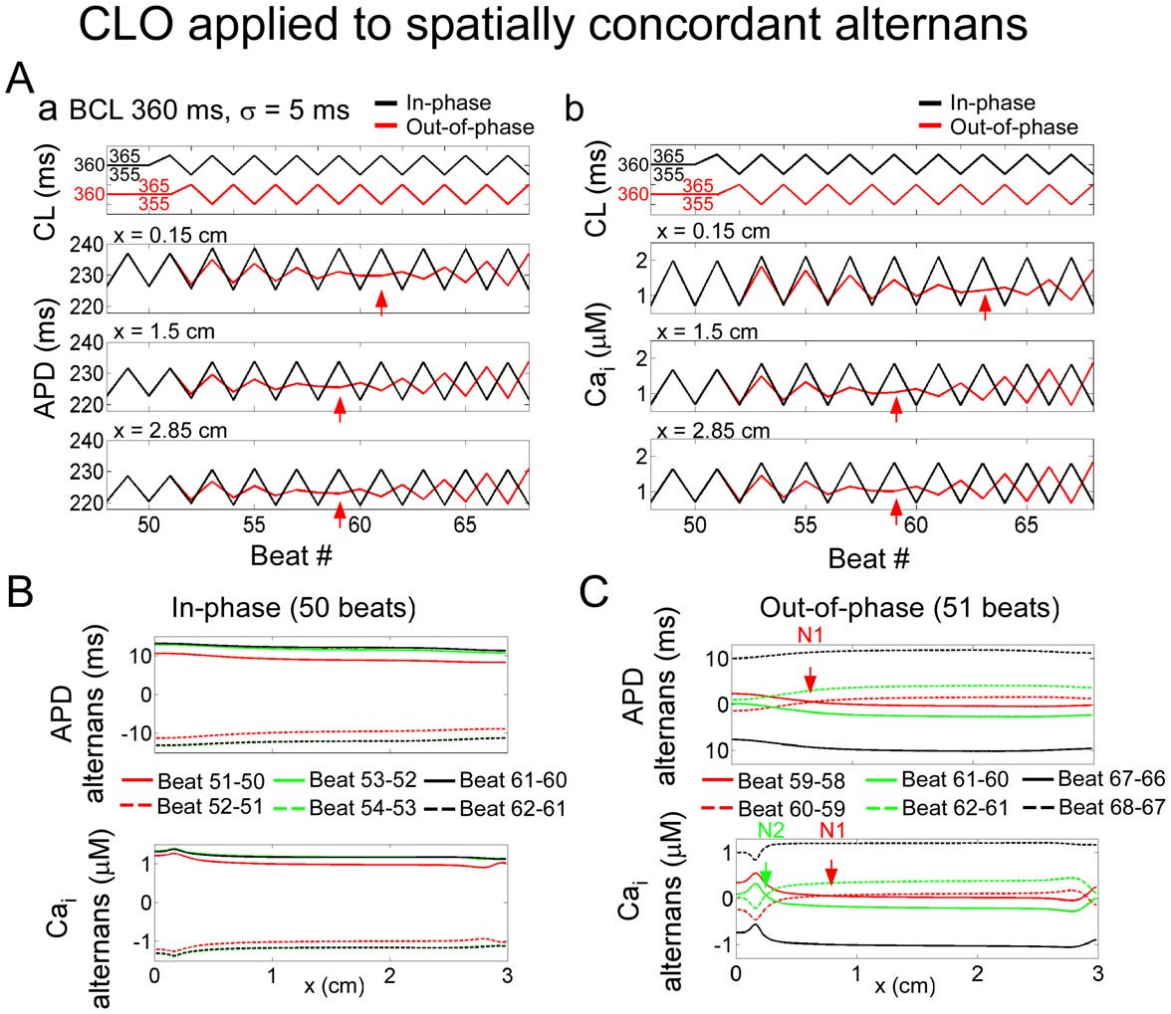
Cycle length oscillation applied during spatially concordant alternans in a one-dimensional cable ionic model. Simulation results are for the condition in which constant pacing at 360 ms induced spatially concordant alternans (*u* = 10 s^−1^). **A.** CLO applied when alternans induced near the pacing site was in-phase (black traces) and out-of-phase (red traces). (a) APD and (b) Ca_i_ transient amplitude measurements as a function of beat number at locations near the pacing site (x = 0.15 cm, top panels), center of the cable (x = 1.5 cm, middle), and end of the cable (x = 2.85 cm, bottom). CL is shown for reference above each set of plots. Phase reversal is indicated by a red arrow. **B, C.** APD (top) and Ca_i_ (bottom) alternans amplitudes along the length of the cable for sequential beat pairs, for CLO initially in-phase (after 50 beats) (**B**) or out-of-phase (after 51 beats) (**C**) with the alternans at the pacing site. Traces for successive beat pairs have the same shading. Nodes (N) are indicated and numbered in order of occurrence.

As in the single myocyte, CLO induced APD and Ca_i_ alternans along a one-dimensional cable (sample V_m_ and Ca_i_ traces are shown in Fig. S1). If CLO was applied to a cable in which spatially concordant alternans was already present following constant pacing, CLO could transiently convert the spatial concordance into discordance. For example, constant pacing at *BCL* of 360 ms induced spatial concordance in our model. Subsequent application of CLO (*σ* = 5 ms = 1.4% *BCL*) produced one of two outcomes. If the first “long” CL of CLO was applied in-phase, CLO augmented the alternans amplitude, as in the single cell, along the entire cable and did not affect spatial concordance (Fig. 2A a, b black traces, B).

If the first “long” CL of CLO was applied out-of-phase (Fig. 2A a, b red traces, C), a phase-reversal (indicated by red arrows) occurred, as with the single cell, along the cable and transiently induced spatial discordance. The phase-reversal first occurred away from the pacing site (Fig. 2A a, b middle, bottom panels), and later near the pacing site (top panels). Alternans became spatially discordant, with a node that formed near the middle of the cable, and then moved towards the pacing site over successive beats (Fig. 2C red, green traces). As in the single cell simulations, the APD and Ca_i_ phase reversals did not occur on the same beat in all locations, resulting in transient electromechanical discordant alternans (Fig. 2A a, b). Eventually spatial concordance was reestablished along the entire cable, with the same steady-state alternans amplitude regardless of the initial phase, as in the single cell simulations (Fig. 2C black traces).

**Figure 3:**
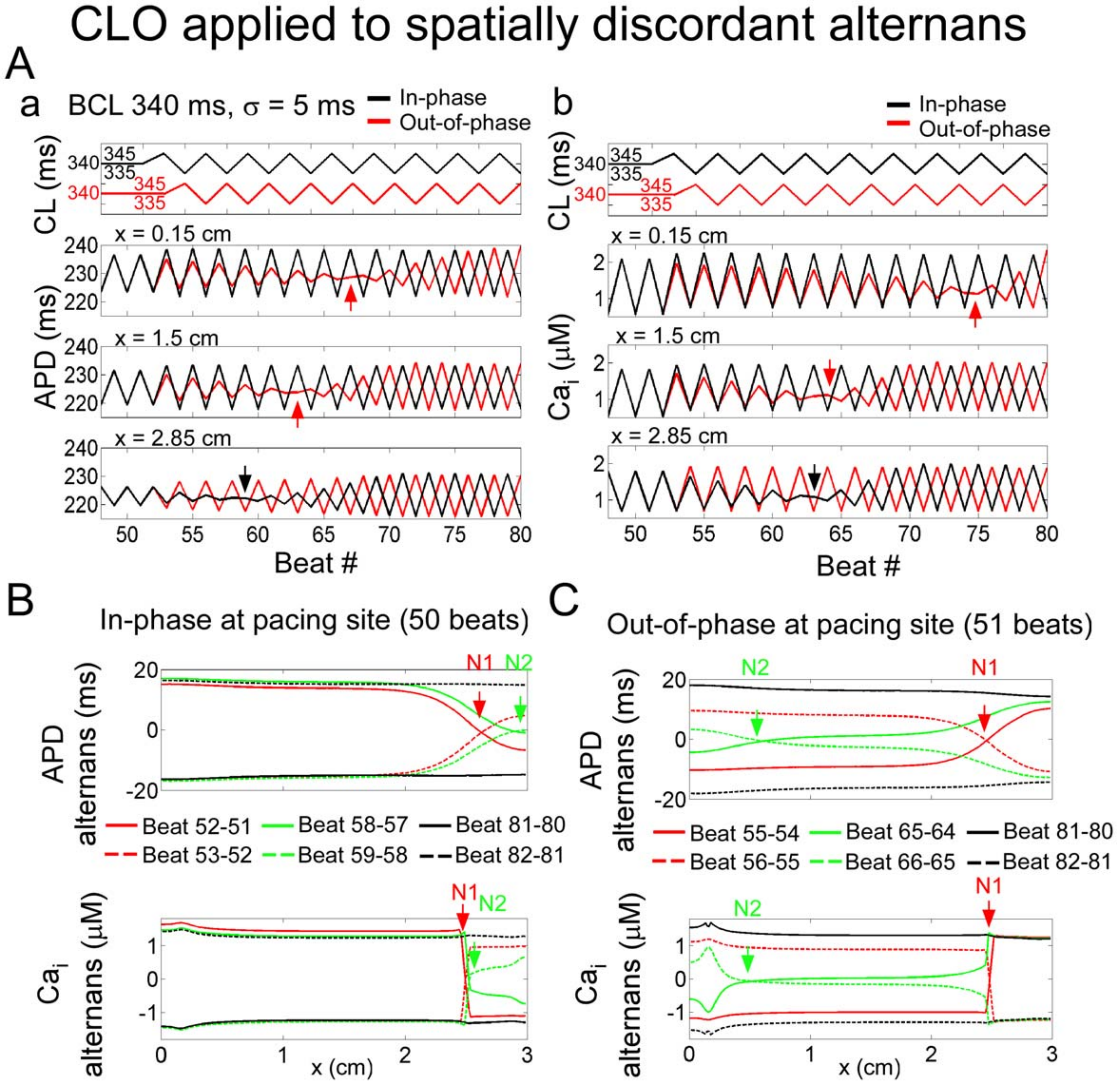
Cycle length oscillation applied during spatially discordant alternans in a one-dimensional cable ionic model. Simulation results are for the condition in which constant pacing at 340 ms induced spatially concordant alternans. See Figure 2 legend for description of the panels. Phase reversals are indicated by a black or red arrow.

If CLO was applied to a cable in which spatially discordant alternans and a node were already present following constant pacing (e.g., with *BCL* = 340 ms; *σ* = 5 ms = 1.5% *BCL*), “phase-matching” occurred along the cable, in which regions of the cable out-of-phase with the applied CLO reversed phase. When CLO and APD alternation at the pacing site were initially *in-phase*, the phase-reversal occurred in the *out-of-phase* region of the cable, distal to the node (Fig. 3A a, b black traces, bottom panels, black arrows). As a consequence, the node location moved farther away from the pacing site (Fig. 3B red, green traces) until the entire cable was spatially concordant (Fig. 3B, black traces).

When CLO and APD alternation at the pacing site were initially *out-of-phase*, the phase-reversal occurred in the region proximal to the node (Fig. 3A a, b red traces, top and middle panels, red arrows), causing the node to move towards the pacing site (Fig. 3C red, green traces) until the entire cable was spatially concordant (Fig. 3C black traces). In both cases, while also transiently inducing electromechanical discordant alternans, CLO ultimately induced the same steady-state spatial concordance regardless of the initial phase, as in the previous examples (Fig. 3A a, b).

### Influence of spatial scale: cell monolayer experiments

**Figure 4:**
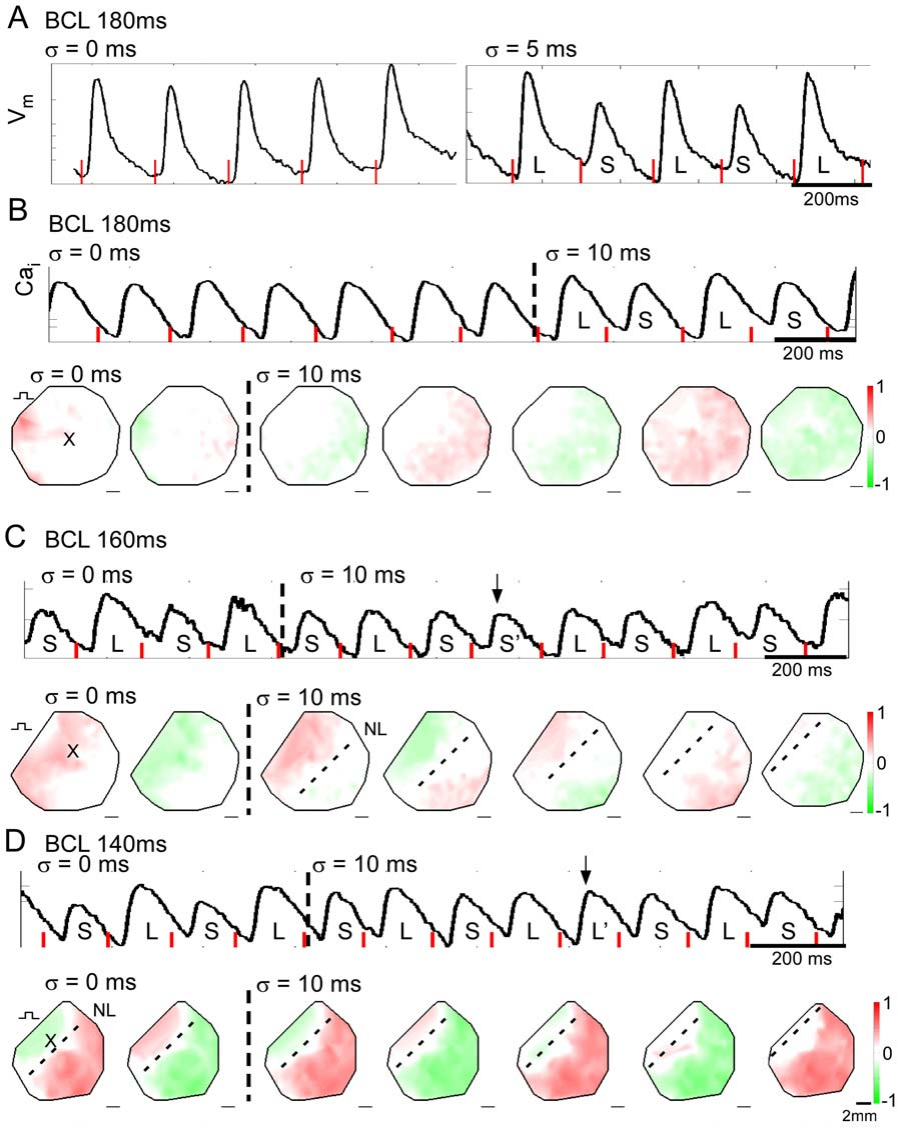
Cycle length oscillation in a cell monolayer. **A.** V_m_ before and during CLO. L and S indicate long and short APDs, respectively. **B**, **C**, and **D.** Ca_i_ traces and successive Ca_i_ alternans maps before and during CLO. Trace is from site indicated by x on the map. L and S indicate large and small amplitude transients, respectively. Red lines indicate timing of stimuli, and pacing site and nodal lines (NL) are indicated by square pulse symbol and dashed lines on alternans maps, respectively. CLO for the case of no or small amplitude alternans at *BCL* = 180 ms (**B**), spatially concordant alternans at *BCL* = 160 ms (**C**), and spatially discordant alternans at *BCL* = 140 ms (**D**) induced by constant pacing prior to CLO (*σ* = 10 ms). Recordings in **B**, **C**, and **D** are from the same monolayer, and **A** is from a different monolayer. Phase reversals are indicated by the black arrows.

We next performed optical mapping of cardiac tissue to investigate the potential proarrhythmic consequences of CLO and to determine if predictions from simulations hold under experimental conditions. Cell monolayers were an ideal choice because they are a simplified homogeneous experimental model, devoid of structural and functional variation that occurs in intact tissue, and the activity of the entire tissue can be measured [21]. Traces from voltage mapping of cell monolayers during constant pacing and CLO are shown in Figure 4A (*BCL* = 180 ms; *σ* = 5 ms = 2.8% *BCL*). In all 8 monolayers tested, no or small amplitude alternans was induced during constant pacing, and CLO induced large amplitude, spatially concordant APD alternans at short *BCL*s. Similarly, in calcium mapping experiments (n = 15 monolayers) in which no or small amplitude alternans was induced during constant pacing at a given *BCL*, CLO induced spatially concordant Ca_i_ alternans, the amplitude of which increased over several beats until reaching a steady-state (*BCL* = 180 ms; *σ* = 10 ms = 5.6% *BCL* in Fig. 4B).

When CLO was applied out-of-phase to a spatially concordant monolayer during voltage mapping experiments, we did not observe any instances of spatially discordant APD alternans. However, during calcium mapping experiments, a transient state of spatial discordance was indeed observed, and the nodal line moved towards the pacing site over successive beats (*BCL* of 160 ms, *σ* = 10 ms = 6.3% *BCL* in Fig. 4C), consistent with the cable simulation in Figure 2C. When CLO was applied to a spatially discordant monolayer, out of phase with the Ca_i_ alternans at the pacing site, there was a gradual transition to spatial concordance as the nodal line moved towards the pacing site (*BCL* = 140 ms; *σ* = 10 ms = 7.1% *BCL* in Fig. 4D), consistent with the cable simulation in Figure 3C. These results were obtained in 7/15 monolayers. In the other 8 monolayers, constant pacing failed to induce Ca_i_ alternans at any of the *BCL*s tested so that the effects of CLO applied during alternans could not be determined. Thus, despite failing to reproduce spatially discordant APD alternans, the cell monolayer experiments generally confirmed the theoretical predictions for induction of transient spatial discordance, movement of nodal lines, and steady-state spatial concordance of Ca_i_ alternans.

### Influence of spatial scale and spatial heterogeneity: whole heart experiments

**Figure 5:**
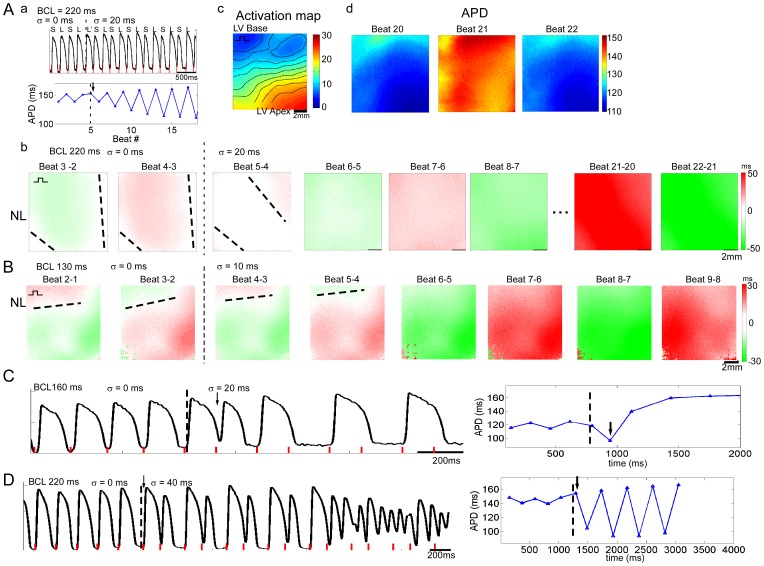
Cycle length oscillation in an isolated adult guinea pig heart. **A.** (a) V_m_ and APD before and during CLO for *BCL* of 220 ms and *σ* = 20 ms. Phase reversal is indicated by the black arrow. (b) Successive APD alternans maps before and during CLO for low amplitude, spatially concordant alternans. Nodal lines (NL) are indicated by dashed lines. (c) Activation map. Pacing site near the left ventricular (LV) base indicated by square pulse symbol. (d) Successive APD maps after reaching steady-state. **B.** Successive APD alternans maps before and during CLO for spatially discordant alternans induced prior to CLO at *BCL* = 180 ms. **C, D.** V_m_ and APD before and during CLO that resulted in loss of 1∶1 capture after several beats (**C**) and initiated ventricular fibrillation (**D**). Red lines indicate timing of stimuli. Recordings in **A** and **D** are from same isolated heart, and **B** and **C** are from a different heart.

Finally, we investigated CLO in isolated adult guinea pig hearts utilizing voltage mapping (n = 5) to determine if observations from the simplified models held in the intact heart. We first confirmed the simulation and cell monolayer results for CLO applied during spatially concordant and discordant APD alternans. As in the previous examples, CLO that was initially out-of-phase with a spatially concordant heart induced a phase-reversal, such that nodal lines in general moved towards the pacing site (*BCL* = 220 ms; *σ* = 20 ms = 9.1% *BCL* in Fig. 5A a, b). However the spatial pattern was more complex when compared with the simulation and cell monolayer results, as the phase-reversal created a wide nodal region as opposed to a line near the pacing site. As in the cable simulation and cell monolayer, spatial concordance was subsequently reestablished, with larger amplitude alternans compared with constant pacing. CLO also increased the amplitude of alternation and induced phase-reversals in the computed pseudo-ECG, a waveform allowing a clinical interpretation of the electrical activity in the isolated heart (Text S1, Fig. S2).

CLO applied to a spatially discordant heart, out-of-phase with the APD alternans induced at the pacing site during constant pacing, caused the nodal line to move towards the pacing site until a state of spatial concordance (*BCL* of 130 ms; *σ* = 10 ms = 7.7% *BCL* in Fig. 5B), consistent with the simulation (Fig. 3C) and cell monolayer (Fig. 4D) results. Increasing *σ* resulted in a greater increase in alternans amplitude, such that it was possible to lose 1∶1 capture (*BCL* of 160 ms, *σ* = 20 ms = 12.5% *BCL* in Fig. 5C). In 3 out of 5 isolated hearts, while CLO was applied near the minimum CL (*MCL*) that maintained capture, induction of alternans was followed by initiation of ventricular fibrillation, as shown in Fig. 5D (*BCL* of 220 ms, *σ* = 40 ms = 18.2% *BCL*), compared with fibrillation initiation in 1 out of 5 hearts during constant pacing (*P* = 0.48). Constant pacing even at the shorter of the two oscillating cycle lengths used during CLO (*BCL*−*σ* = 180 ms in Fig. 5D) did not initiate fibrillation, although alternans was observed. Significantly, in all instances of ventricular fibrillation initiated during CLO, *σ* was large (19.9±2.3%, *n* = 4 instances), and *BCL*−*σ* was within 20 ms of *MCL* (13.8±7.5 ms). Thus, the isolated heart experiments further confirmed the simulation predictions regarding spatial concordance and illustrated that in some cases, CLO could initiate ventricular fibrillation.

## Discussion

In this study, we demonstrated that CLO can transiently promote a proarrhythmic substrate while also promoting an antiarrhythmic, steady-state condition across multiple models, species, and spatial scales (Table 1). We demonstrated that CLO imposes a particular phase to the alternation, such that at steady-state the “long” (or “short”) APD always follows the “long” (or “short”) CL, and the phase of APD alternation is in sync with the phase of CLO in both the single cell and tissue. In essence, the “system” is being driven into an in-phase condition. However, the transition towards this in-phase condition includes a transient out-of-phase condition, at both the cellular level by inducing electromechnical discordance and at the tissue level by inducing spatial discordance in voltage and calcium. Our cell monolayer and isolated heart experiments validated the simulation predictions of both transient spatial discordance and steady-state spatial concordance.

**Table 1:** Summary of the transient response to CLO in different models.

Model	Constant Pacing	CLO applied in-phase	CLO applied out-of-phase
Single cell (0D)	SCA	Increased alternans amplitude	Transient electromechanical discordance
Cable (1D)	SCA	Increased alternans amplitude	Transient spatial, electromechanical discordance; node progressed towards pacing site until SCA
	SDA	Node progressed away from pacing site until SCA	Node progressed towards pacing site until SCA
Cell monolayer (2D)*	SCA	Increased alternans amplitude	Transient spatial discordance; nodal line progressed towards pacing site until SCA
	SDA	(not observed)	Nodal line progressed towards pacing site until SCA
Isolated heart (3D)	SCA	Increased alternans amplitude	Transient complex spatial discordance pattern until SCA
	SDA	(not observed)	Node progressed towards pacing site until SCA

The responses to CLO applied in and out of phase in each model are shown during initial states of spatially concordant alternans (SCA) and spatially discordant alternans (SDA).

* Cell monolayer responses refer to those observed during calcium mapping experiments.

Previous studies by Gauthier and colleagues demonstrated that CLO induced and amplified alternans in an iterative map model of APD restitution and in the frog heart [15]–[17]. Other studies have shown that amplification following applied repetitive perturbations is in fact a universal phenomenon observed in many dynamical systems, specifically near a bifurcation point (e.g. CL of alternans onset in cardiac dynamics) [22], [23]). Our study confirmed this amplification in mammalian cardiac cells and tissue but also revealed novel properties and consequences of CLO perturbations, as described above.

A similar phenomenon has been observed in subcellular calcium dynamics, in which CLO applied to subcellular discordant Ca_i_ alternans imposes “phase-matching,” which causes nodes of zero alternans to drift towards the cell boundaries [24]. A previous study in isolated myocytes demonstrated that a single premature or delayed beat can sometimes suffice to induce a “phase-reversal” in APD and mechanical alternans [25]. Our study demonstrates that in cardiac tissue, CLO, which in essence is a series of small premature and delayed beats, can induce a phase-reversal after a several beat delay. Our results show that the beat delay differed for voltage and calcium, and also varied at different spatial locations. As a consequence, transient states of spatial and electromechanical discordant alternans were induced until states of concordance were reestablished.

In our study, we observed instances of transient, spatially discordant Ca_i_ but not APD alternans. This observation is in keeping with our previous findings that spatially discordant APD alternans occurred much less frequently than spatially discordant Ca_i_ alternans in neonatal rat ventricular myocyte monolayers [26], which we speculate is related to offsetting effects on APD of calcium-dependent effects on inward and outward ionic currents. Importantly, our mapping experiments of Ca_i_ alternans confirmed predictions of the cable simulations regarding transient spatial discordance and nodal line movement.

Also, our experiments in the isolated heart confirmed predictions from the homogeneous cable simulations and cell monolayer experiments regarding the ability of CLO to perturb spatially concordant alternans – transiently inducing spatially discordant alternans, causing nodal line movement, and reestablishing steady-state spatial concordance. However, CLO effects on spatial discordance were more complex in the isolated heart (Fig. 5A b). It is known that conduction velocity restitution (the dependence of conduction velocity on DI) can induce both spatially discordant alternans and CLO [27], and that spatial heterogeneity in APD (Fig. 5A d) and APD restitution augment alternans induction [4]. Further investigation is needed to systematically investigate whether and how conduction velocity restitution and heterogeneous APD restitution, which we did not explicitly examine in this study, may augment the effects of CLO in terms of electromechanical and spatial discordance.

If CLO is applied “out-of-phase” to spatially concordant alternans, spatial discordance – a more proarrhythmic condition [2] – is induced transiently but eventually spatial concordance is reestablished. Significantly, if CLO is applied to spatially discordant alternans, spatially concordant alternans eventually results after several beats, regardless of the initial phase. The conversion of spatially discordant alternans to concordant alternans is generally considered to be antiarrhythmic in homogeneous substrates. In our study, although not to the level of significance, we did observe instances of ventricular fibrillation. We speculate fibrillation initiation may have occurred as a consequence of the heterogeneous conditions present in the intact heart. In particular, since fibrillation initiation occurred during CLO with large amplitude *σ* and *BCL*−*σ* near *MCL*, the large amplitude CLO will dramatically shorten the DIs, more so in a region of intrinsically longer action potentials. As DI shortens, conduction will slow, increasing the dispersion of repolarization and therefore the likelihood of local conduction block [2]. Although our study shows CLO can have proarrhythmic consequences under certain conditions, we found that CLO established spatial concordance under all conditions. Future studies are needed to investigate the use of CLO as a pacing therapy to terminate spatially discordant alternans and test if the potential benefit of CLO at steady-state outweighs the transient proarrhythmic consequences.

### Limitations

A limitation of this study is that no single species and model has been developed that can address all the questions posed in this study at the different spatial scales and degrees of complexity. Thus, different but appropriate models were chosen that could dissect out the varying effects of CLO at the cellular and tissue levels. These models are widely used and well-characterized, and all have been previously utilized to study the dynamics of alternans and arrhythmogenesis [3]–[5], [26]. Importantly, the consistency between models, species, and spatial scales demonstrated the generality of the response to CLO in the heart, and the simulations accurately predicted and explained the qualitative behavior of the experiments. However, extrapolation of these results to the human remains to be verified, given the species differences of rat, guinea pig, and dog in terms of their electrophysiology and calcium cycling.

In several previous clinical studies, sequences of CLO as brief as only a few heart beats (“short-long-short” sequence) in heart rate can be seen immediately preceding the onset of an arrhythmia [28], [29]. Although we did not directly examine the consequences of such short sequences, our results show that APD and Ca_i_ alternans amplitude increased until reaching a steady-state, occurring after many beats. Further studies are needed to evaluate the role of CLO duration.

## Supporting Information

Text S1 **Supporting information, including detailed methods, supplemental results, and supplemental references.**
(DOC)

Figure S1 **Cycle length oscillation in a one-dimensional cable ionic model.** V_m_ and Ca_i_ before and during CLO along the length of the cable. Traces from the pacing site (x=0 cm) are the lowermost of the stacked traces. L and S indicate long and short APDs and large and small Ca_i_ transient amplitudes, respectively.
(TIF)

Figure S2 **Alternans in the pseudo-ECG.** **A.** V_m_ and pseudo-ECG (pECG) before and during CLO. Red lines indicate timing of point stimuli. At the top, pECG is shown on an expanded time scale, and the QRS complex, T wave, QT interval, and RR interval are identified. **B.** CL, RR interval, QT interval, R wave magnitude, and T wave magnitude plotted as a function of time. Black arrows indicated phase reversals.
(TIF)
