# Supplementary material for: Oscillation in Cycle Length Induces Transient Discordant and Steady-State Concordant Alternans in the Heart
Source: PLoS One. 2012 Jul 5;7(7):e40477. doi: 10.1371/journal.pone.0040477 (PMC3390356; doi:10.1371/journal.pone.0040477)
Supplement: Text S1 — Supporting information, including detailed methods, supplemental results, and supplemental references. (DOC) [file pone.0040477.s001.doc]

**SUPPORTING INFORMATION**

**Detailed Methods**

*Ionic Model Simulations*

Single cell simulations were performed using the Shiferaw-Sato-Karma ionic model [1], which integrates a detailed description of calcium cycling with a canine ionic model. One-dimensional cable simulations were simulated by the following partial differential equation:

$\frac{\partial V_{m}}{\partial t}=-(I_{ion}+I_{stim})/C_{m}+D\frac{\partial^{2}V_{m}}{\partial x^{2}}$, [1]

where V_m_ is the transmembrane potential, I_ion_ is the total ionic current described by the ionic model, I_stim_ is the applied stimulus current, C_m_ is the membrane capacitance (1 μF/cm^2^), and D is the diffusion coefficient (0.5 cm^2^/s). The cable simulation was solved numerically, using a forward Euler method with a time step of 1 μs and spatial step of 150 μm. The cable length was set to 3 cm (200 cells). For both sets of simulations, state variables, including V_m_ and Ca_i_, were output at 10 μs time steps. The time constant of the inactivation gate of the L-type calcium channel (which when increased promotes voltage-driven alternans [1,2]) was set to 35 ms. The sensitivity of sarcoplasmic reticulum (SR) calcium release to SR load, *u* (which when increased promotes calcium-driven alternans [1,2]), was set to 6 or 10 s^-1^ for single cell simulations and to 10 s^-1^ in the cable simulations.

Constant pacing (pacing at a constant CL) was applied to the models for 50 or 51 beats, after which CLO, defined as pacing with CL:

$CL_{n}=BCL+(-1)^{n+1}\sigma$, [2]

was applied for an additional 50 beats. *BCL* is a constant basic cycle length, and *σ* is the amplitude of the applied oscillation, which was typically in the range of 5-40 ms (1-25% of *BCL*). The single cell and cable were paced by 1-ms direct current injection stimulation of 50 and 150 μA/cm^2^, respectively. The cable was paced by stimulating the cells between *x* = 0 and *x* = 0.75 mm.

*Cell Monolayer Experiments*

Our procedure to create and optically map cultured cell monolayers has been previously described [3]. Briefly, neonatal rat ventricular myocytes (NRVMs) were enzymatically dissociated from 2-day old Sprague-Dawley rat hearts, plated at high density onto 21 mm diameter plastic coverslips to form confluent monolayers, and stained with the 10 μM voltage-sensitive fluorescent dye, di-4-ANEPPS, or the 5 μM calcium-sensitive fluorescent dye, Rhod-2-AM, and continually superfused with Tyrode’s solution. Contact fluorescent imaging was used to optically map the cell monolayers. To promote APD or Ca_i_ alternans, cell monolayers were also superfused with 10 μM of the K_ATP_ channel agonist pinacidil, and experiments were performed at room temperature [2].

*Isolated Guinea Pig Heart Experiments*

Our procedure for optically mapping isolated guinea pig hearts has been previously described [4]. Briefly, Hartley guinea pigs (200 – 700 g) were anesthetized with sodium pentobarbitol. After a midline thoracotomy, the hearts were excised, mounted on a Langendorff perfusion system, stained with 10 μM di-4-ANEPPS, and immersed in a transparent plexiglass chamber filled with Tyrode’s solution. A tandem-lens assembly, including two 150 W halogen lamps and a 100 x 100 pixel CMOS camera (Ultima-L, SciMedia, Costa Mesa, CA) was used to optically map the isolated hearts.

*Experimental Protocol*

Cell monolayers or isolated hearts were paced using a platinum bipolar point electrode at a constant cycle length for 50 beats, after which CLO for a given *σ* was applied as described above. Cell monolayers were paced near the cover slip edge, and isolated hearts were paced on the epicardial surface at the left ventricular base. Pacing sites are indicated in the spatial maps presented.

*Data Analysis*

The individual signals recorded during optical mapping were temporally filtered using a 5 point median filter, baseline-corrected by subtraction of a fitted 3^rd^ order polynomial, and range normalized. Experimental and simulated APD measurements were computed as the time of 80% recovery from peak amplitude. Ca_i_ and APD alternans maps were computed by taking the difference of Ca_i_ transient amplitudes and APDs, respectively, on successive beats at each recording site for experiments or discretized location for cable simulations. Nodes and nodal lines were identified as regions of no alternans. The bipolar pseudo-electrocardiogram (pseudo-ECG) was computed as previously described [5].

*Statistical Analysis*

Summary values were expressed as mean ± standard deviation. Statistical significance for paired comparisons of instances of ventricular fibrillation was determined using McNemar’s Chi-squared test.

**Supplemental Results**

*Alternans in cable simulations*

Representative voltage and calcium traces along the length of the cable before and during CLO are shown in Figure S1. Small amplitude alternans is induced during constant CL pacing. As in the single cell simulations, CLO induced large APD and Ca_i_ alternans, growing in amplitude over several beats.

*Alternans in the pseudo-ECG*

The pseudo-ECG provides a waveform for a clinical interpretation of the electrical activity of an isolated heart during CLO. The QRS complex and T wave could be clearly identified during each electrical wave (Fig. S2A top). Since only epicardial ventricular electrical activity was optically mapped, the P wave was not present and the T wave was inverted, due to the absence of atrial and sub-epicardial electrical activity, respectively. As expected, the rapid R wave corresponded with the action potential upstroke, and the T wave corresponded with the action potential repolarization (Fig. S2A). In this example, constant CL pacing induced spatially concordant alternans (Fig. 5A of main text). The pseudo-ECG showed alternation in the QT interval, as well as in the magnitude of the R and T waves (Fig. S2A, B). The “large” R wave and the “small” T wave accompanied the “long” APD and “long” QT interval. CLO induced a phase-reversal in the QT interval and R and T wave magnitude (Fig. S2B, black arrows), followed by larger amplitude alternation, compared with constant CL pacing.

**Supplemental References**

1. Shiferaw Y, Sato D, Karma A (2005) Coupled dynamics of voltage and calcium in paced cardiac cells. Phys Rev E Stat Nonlin Soft Matter Phys 71: 021903.

2. Weinberg S, Malhotra N, Tung L (2010) Vulnerable windows define susceptibility to alternans and spatial discordance. Am J Physiol Heart Circ Physiol 298: H1727-1737.

3. Weinberg S, Lipke EA, Tung L (2010) In vitro electrophysiological mapping of stem cells. Methods Mol Biol 660: 215-237.

4. Tandri H, Weinberg SH, Chang KC, Zhu R, Trayanova NA, et al. (2011) Reversible cardiac conduction block and defibrillation with high-frequency electric field. Sci Transl Med 3: 102ra196.

5. Weinberg S, Iravanian S, Tung L (2008) Representation of collective electrical behavior of cardiac cell sheets. Biophys J 95: 1138-1150.
