# Supplementary figures and images for: Oscillation in Cycle Length Induces Transient Discordant and Steady-State Concordant Alternans in the Heart
Source: PLoS One. 2012 Jul 5;7(7):e40477. doi: 10.1371/journal.pone.0040477 (PMC3390356; doi:10.1371/journal.pone.0040477)

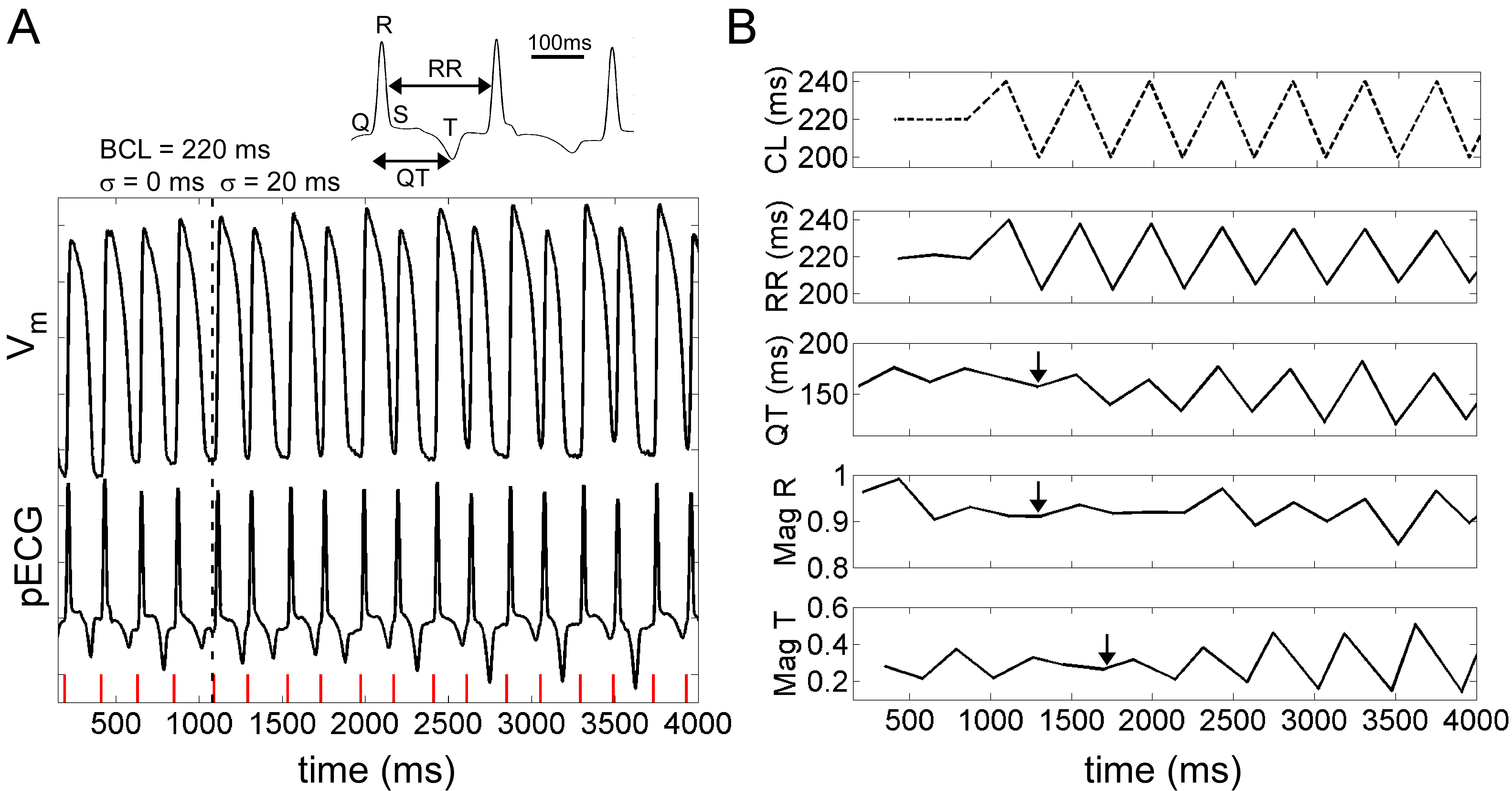

Supplement: Figure S2 — Alternans in the pseudo-ECG. A. Vm and pseudo-ECG (pECG) before and during CLO. Red lines indicate timing of point stimuli. At the top, pECG is shown on an expanded time scale, and the QRS complex, T wave, QT interval, and RR interval are identified. B. CL, RR interval, QT interval, R wave magnitude, and T wave magnitude plotted as a function of time. Black arrows indicated phase reversals. (TIF) [file pone.0040477.s003.tif]
